# Supplementary material for: Collagen VI microfibril structure reveals mechanism for molecular assembly and clustering of inherited pathogenic mutations
Source: Nat Commun. 2025 Aug 14;16:7549. doi: 10.1038/s41467-025-62923-3 (PMC12354898; doi:10.1038/s41467-025-62923-3)
Supplement: Supplementary file 1 — Supplementary Information [file 41467_2025_62923_MOESM1_ESM.pdf]

**Collagen VI microfibril structure reveals mechanism for molecular assembly and clustering of inherited pathogenic mutations**

Alan RF Godwin[1], Mark H Becker[1+], Rana Dajani[1+], Matthew Snee[1+], Alan M Roseman[2] and Clair Baldock[1*]

**Supplementary Information**

Supplementary Figure 1: Expression of recombinant mini-collagen VI C-terminal constructs
Supplementary Figure 2: AlphaFold Multimer prediction of heterotrimeric mini collagen VI $\alpha1\alpha2\alpha3^{C1C2}$ construct
Supplementary Figure 3: CryoEM data processing workflow for mini collagen VI $\alpha1\alpha2\alpha3^{C1C2}$
Supplementary Figure 4: CryoEM analysis of heterotrimeric mini collagen VI $\alpha1\alpha2\alpha3^{C1C2}$
Supplementary Figure 5: CryoEM map around the trimeric coiled coil region
Supplementary Figure 6: N-linked glycosylation of heterotrimeric mini collagen VI $\alpha1\alpha2\alpha3^{C1C2}$ and Bovine collagen VI microfibrils.
Supplementary Figure 7: CryoEM data processing workflow for bovine collagen VI microfibril double bead region
Supplementary Figure 8: CryoEM of bovine collagen VI microfibril double bead region
Supplementary Figure 9: CryoEM data processing workflow for bovine collagen VI single bead region
Supplementary Figure 10: CryoEM of the single bead of bovine collagen VI microfibrils
Supplementary Figure 11: CryoEM data processing workflow for the local refinement of the top of the bovine collagen VI microfibril bead region
Supplementary Figure 12: Local refinement of the top of the single bead of bovine collagen VI microfibrils
Supplementary Figure 13: Comparison of collagen VI atomic models
Supplementary Figure 14: Correlation of mini-collagen VI map with microfibril map
Supplementary Figure 15: Docking C1 and N1 domains into microfibril bead map
Supplementary Figure 16: AlphaFold 3 prediction of the heterotrimeric mini collagen VI $\alpha1\alpha2\alpha3^{C1C2}$ construct
Supplementary Figure 17: AlphaFold 3 prediction of the heterotrimeric $\alpha1\alpha2\alpha3^{C1C2}$ region of bovine collagen VI
Supplementary Figure 18: AlphaFold 3 prediction of the heterotrimeric $\alpha1^{N1}\alpha2^{N1}\alpha3^{N1}$ and 100 residues of the collagenous region of bovine collagen VI
Supplementary Table 1: BioSAXS data collection of mini collagen VI $\alpha1\alpha2\alpha3^{C1C2}$ construct
Supplementary Table 2: Cryo-EM data collection, refinement and validation statistics

Uncropped SDS-PAGE gels and western blot shown in figures 2b, 5b, 5f and supplementary figure 1B.

Supplementary Figure 1

**A**

| Construct | Sequence | N-terminal Tag | Molecular weight (Da) | Predicted N-glycans | Notes |
|---|---|---|---|---|---|
| α1α2α3$^{C1}$<br>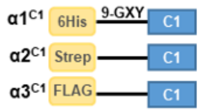<br> | α1: 566 - 828<br>α2: 564 - 832<br>α3: 2347 - 2610 | 6 x His<br>Strep<br>FLAG | 92,872<br>30,401<br>31,814<br>30,657 | 3 potential N-glycans<br>0<br>2 (on C1)<br>1 (on C1) | |
| α1α2α3$^{C1C2}$<br>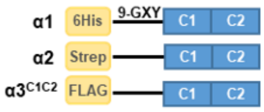<br> | α1 – 566 - 1028<br>α2 – 564 - 1019<br>α3 – 2347 - 2820 | 6 x His<br>Strep<br>FLAG | 158,879<br>52,186<br>52,516<br>54,177 | 9 potential N-glycans<br>2 (on C2)<br>4 (2 on C1, 2 on C2)<br>3 (1 on C1, 2 on C2) | α3 furin site - $^{2607}$RDRR$^{2610}$ to RDAA |
| α1α2α3$^{FL}$<br>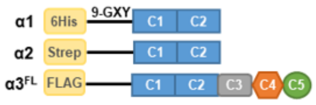<br> | α1 – 566 - 1028<br>α2 – 564 - 1019<br>α3 – 2347 - 3177 | 6 x His<br>Strep<br>FLAG | 197,117<br>52,186<br>52,516<br>92,415 | 10 potential N-glycans<br>2 (on C2)<br>4 (2 on C1, 2 on C2)<br>4 (1 on C1, 2 on C2, 1 on C4) | α3 furin site - $^{2607}$RDRR$^{2610}$ to RDAA |

**B**

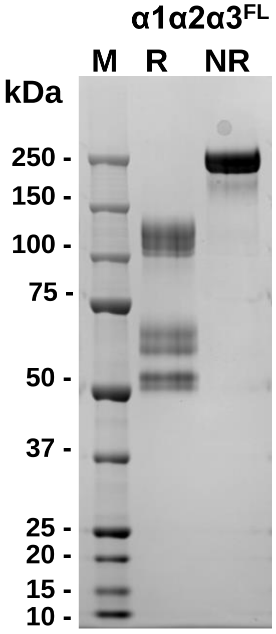


**Supplementary Figure 1 : Expression of recombinant mini-collagen VI C-terminal constructs** (A) Table including details of the human α1α2α3$^{C1}$, α1α2α3$^{C1C2}$ and α1α2α3$^{FL}$ mini-collagen VI constructs generated using sequences from Uniprot human COL6A1 P12109; human COL6A2 P12110; human COL6A3 P12111. (B) Coomassie stained SDS-PAGE gel of the purified α1α2α3$^{FL}$ heterotrimer under reduced (R) and non-reduced (NR) conditions. M is the molecular weight marker. The trimer dissociates into the individual α-chains upon reduction of intermolecular disulphide bonds. Purifications were repeated at least three times with similar results.

Supplementary Figure 2

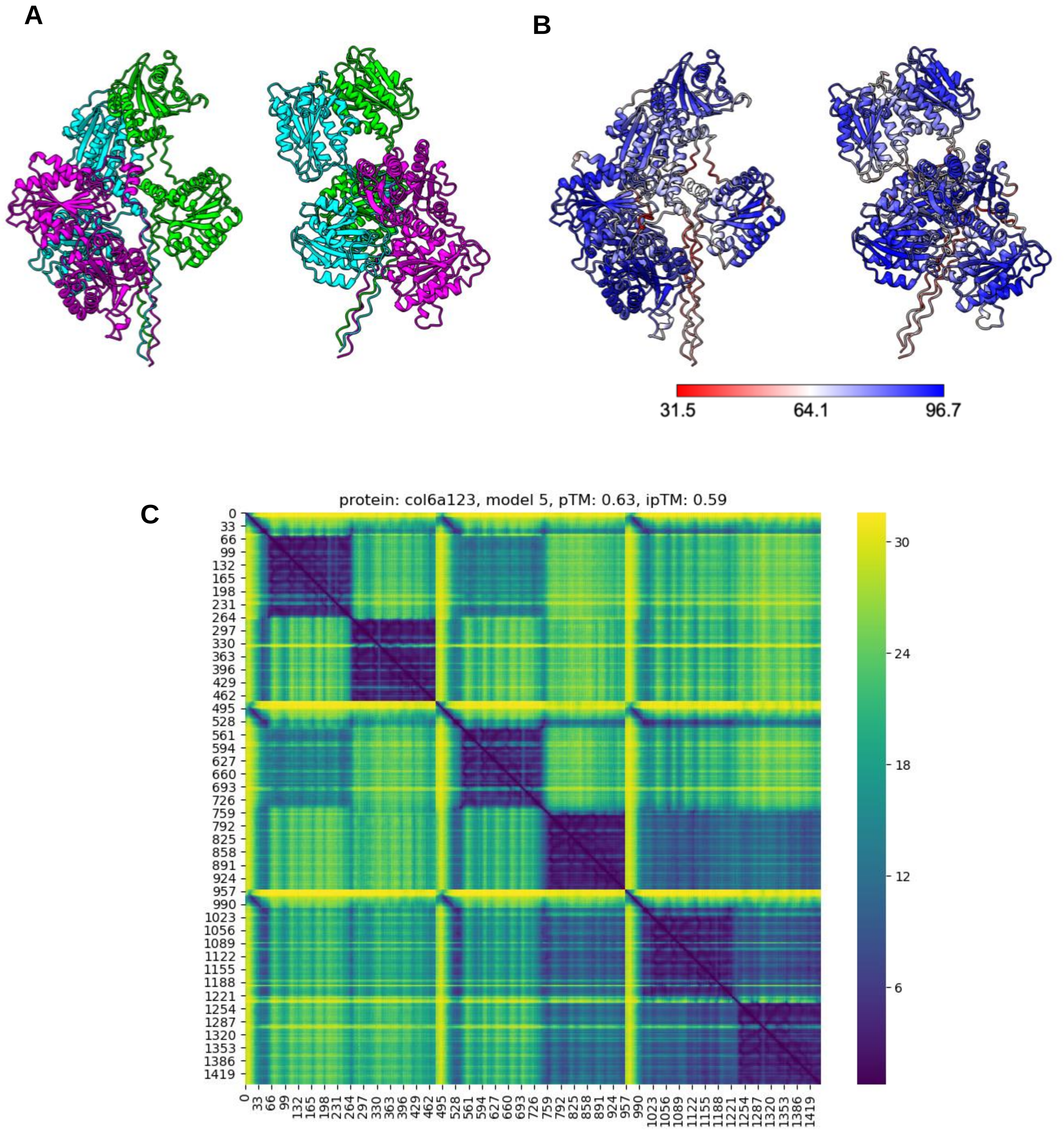


**Supplementary Figure 2 : AlphaFold Multimer prediction of heterotrimeric mini collagen VI α1α2α3$^{C1C2}$ construct** (A) An AlphaFold model of the human α1α2α3$^{C1C2}$ construct. The model was generated based on the sequences used for protein expression as described in Supplementary Figure 1A using AlphaFold 2.1.1. The model is colored by chain α1= green, α2 =cyan and α3 = magenta. (B) The model colored by plDDT score. (C) Predicted alignment error plot coloured by expected position error (Ängströms).

Supplementary Figure 3

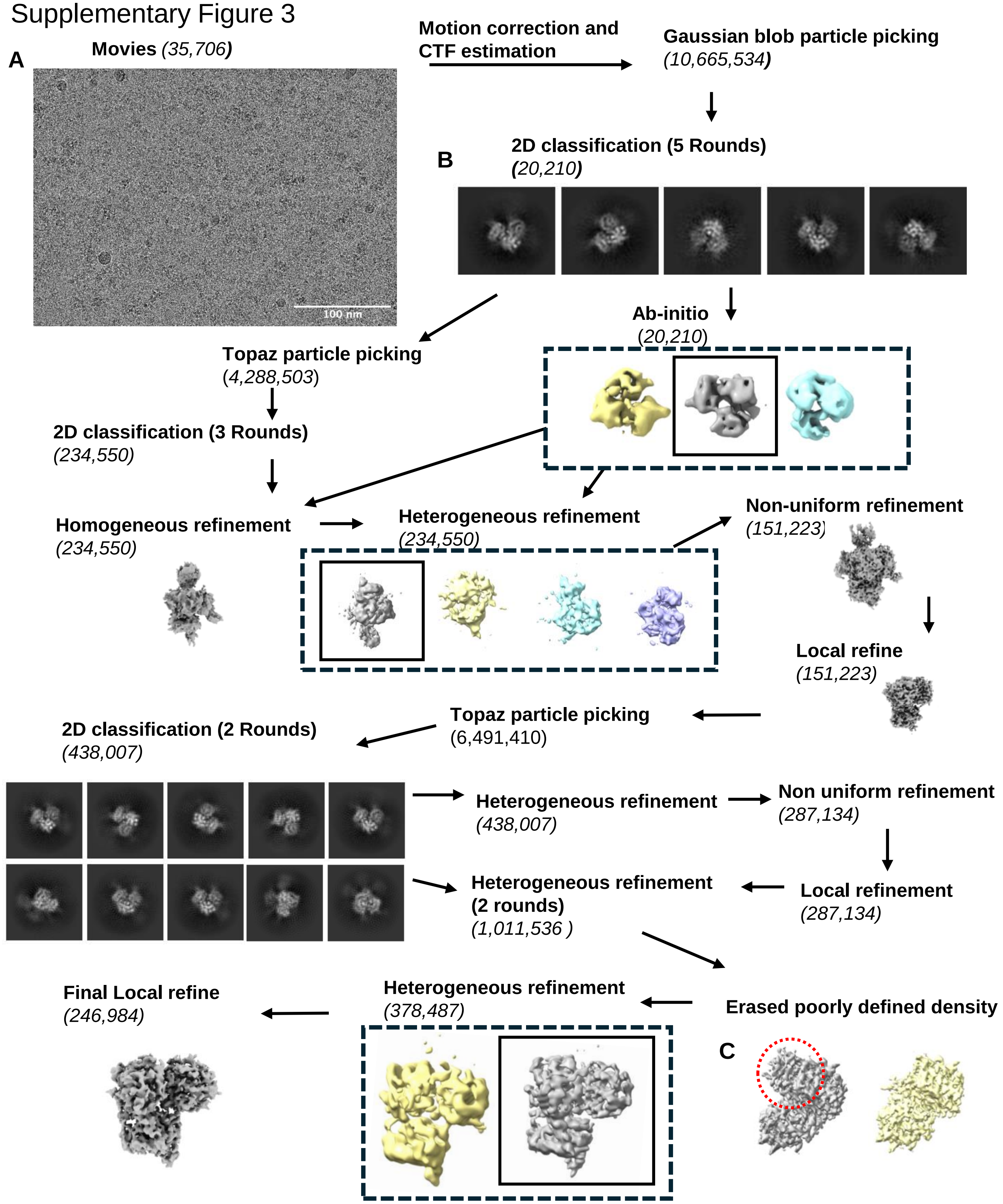


**Supplementary Figure 3: CryoEM workflow for heterotrimeric mini collagen VI α1α2α3$^{C1C2}$ construct.** A schematic diagram showing the cryoEM single particle averaging work-flow for the heterotrimeric mini collagen VI α1α2α3$^{C1C2}$ construct. Numbers of particles used in each step are shown. Black boxes highlight which 3D class was picked in the ab-initio or heterogeneous refinement jobs. (A) Representative cryoEM image of α1α2α3$^{C1C2}$ heterotrimer. Scale bar = 100 nm. (B) Classum images of aligned heterotrimers. The box size is 26 nm X 26 nm. (C) Images of a 3D density map with and without an area of less well resolved density this was removed using Chimera map eraser. The erased area is highlighted with a red dotted circle.

# Supplementary Figure 4

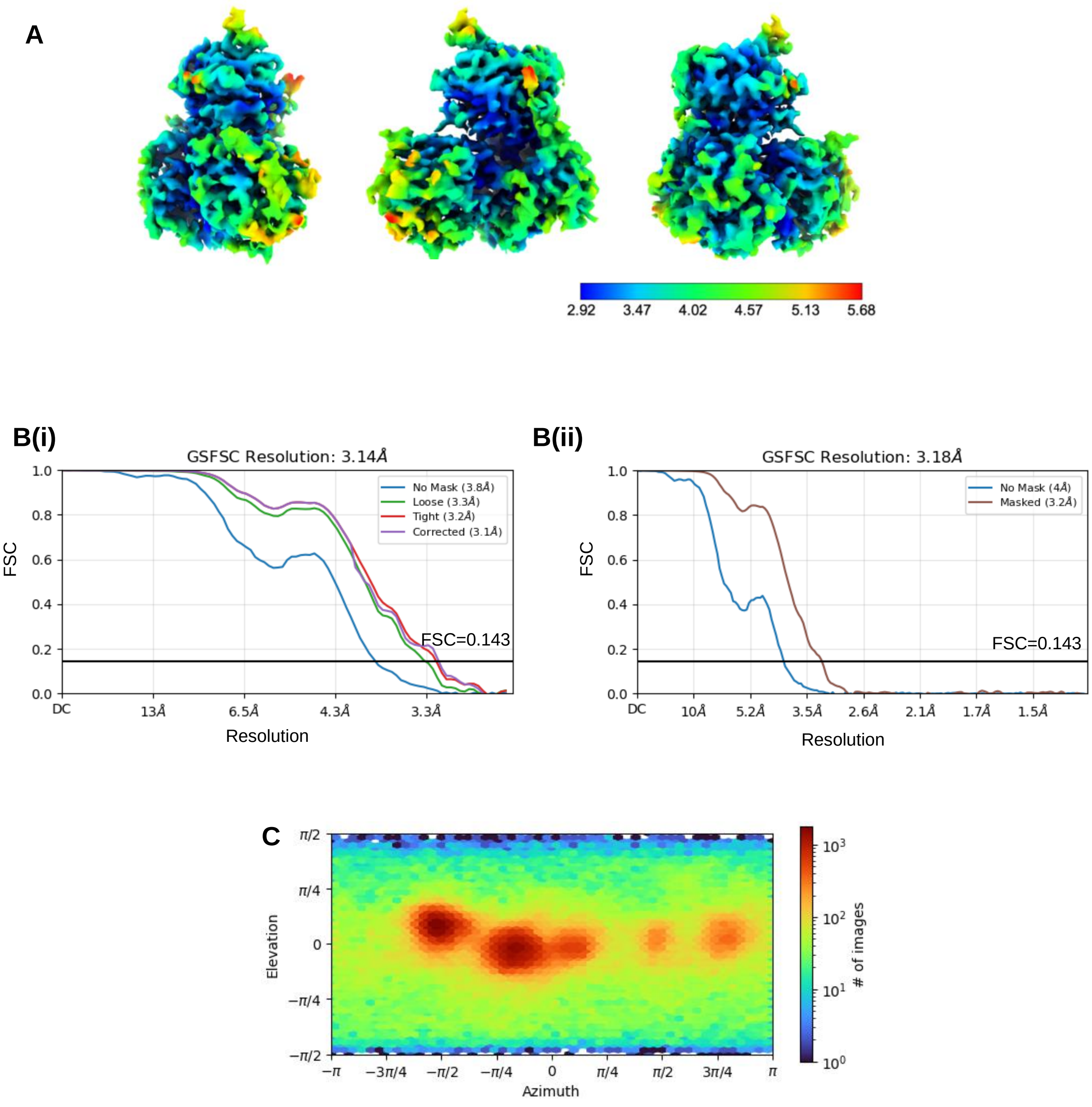


**Supplementary Figure 4: CryoEM analysis of heterotrimeric mini collagen VI α1α2α3$^{C1C2}$ construct**

(A) CryoEM density map of the mini collagen VI α1α2α3$^{C1C2}$ construct coloured by local resolution. The scale is in Angstroms. (B) Plots of the Fourier Shell Correlation (FSC) between two independently refined half-maps of the α1α2α3$^{C1C2}$ heterotrimer. (Bi) The final map was reconstructed from particles which were extracted in a 400 x 400 pixel box and were down-sampled to 200 x 200 pixels with a pixel size of 1.3 Å/pixel. The resolution is 3.14 Å at a cutoff of 0.143. The plot extends out to 2.6 Å, the Nyquist frequency. (Bii) FSC plot of the final refinement without down-sampling, extending out to the Nyquist frequency of 1.3 Å. Particles were extracted in a 400 x 400 box with a pixel size of 0.65 Å/pixel, and were used in a reconstruction using particle alignments from the final binned reconstruction. Source data are provided as a Source Data file. (C) The viewing direction distribution plot of the aligned particles in the final dataset.

Supplementary Figure 5

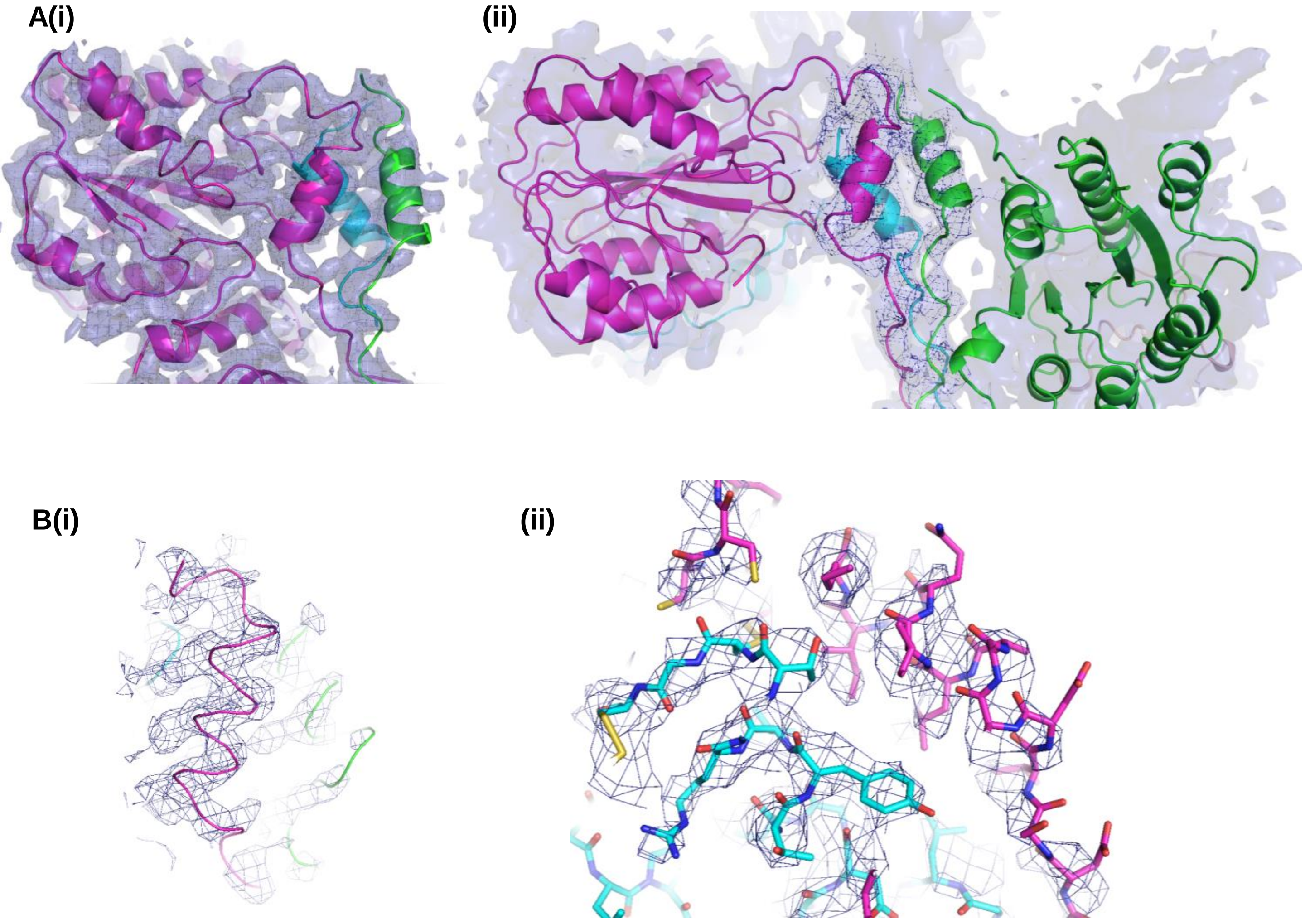


**Supplementary Figure 5**: **CryoEM map around the trimeric coiled coil region**
(A) CryoEM map showing the trimeric coiled coil region for the mini-collagen (i) and bovine microfibril (ii) with the respective models overlaid. (B) The grain of the helix can be seen with side chain densities. (ii) Map – model overlay for the α2 and α3 chains, including density for Tyr597 from the α2 chain, the only aromatic residue in the coiled-coil. The chains are colored α1 = green, α2 = cyan and α3 = magenta. Where shown, non-carbon atoms are coloured according to the CPK convention.

Supplementary Figure 6

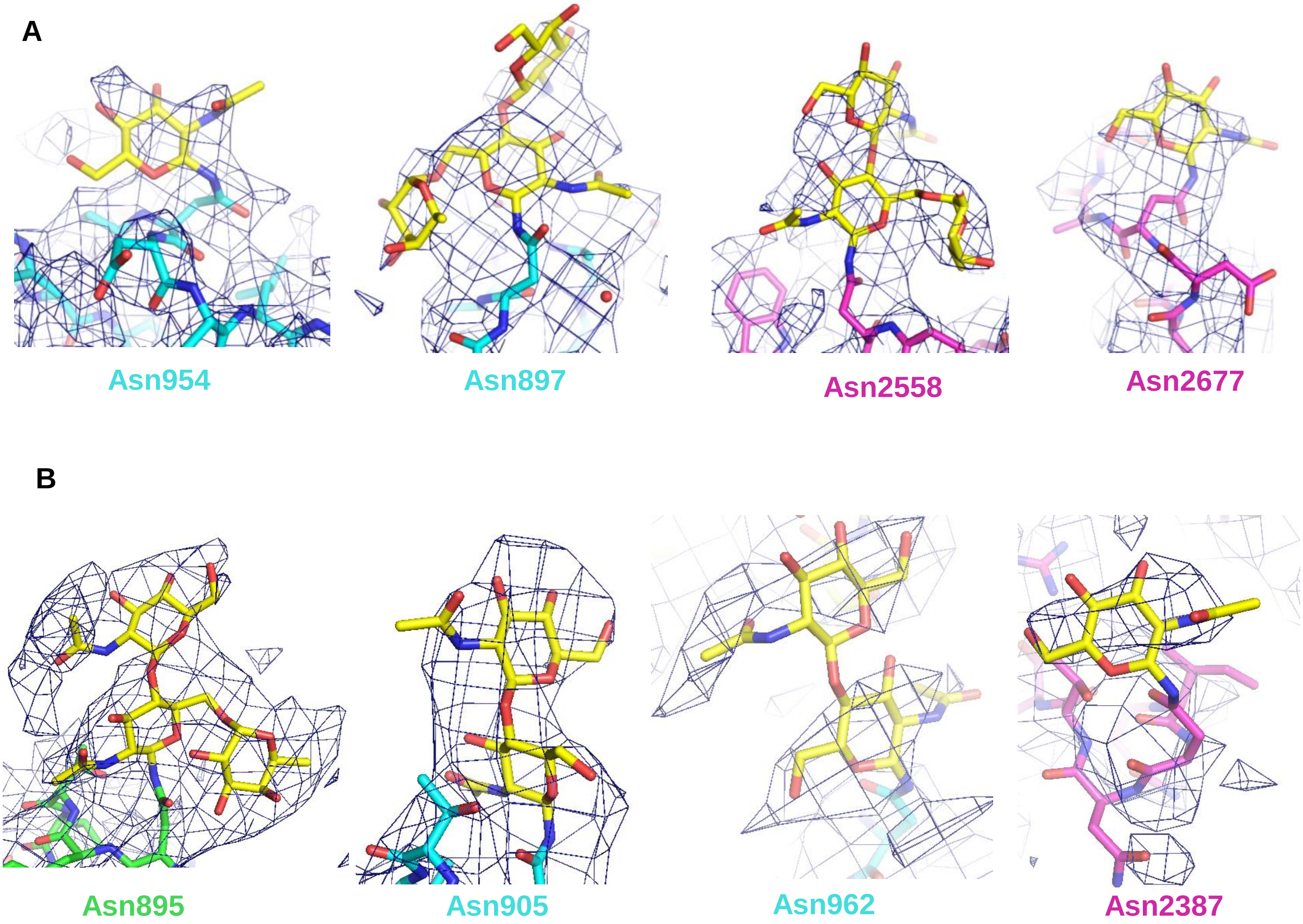


**Supplementary Figure 6: N-linked glycosylation of heterotrimeric mini collagen VI α1α2α3$^{C1C2}$ and bovine collagen VI microfibrils.**

(A) CryoEM density for N-linked glycosylation of asparagine residues in human α1α2α3$^{C1C2}$ heterotrimer and (B) bovine collagen VI microfibrils. Asparagine residues and associated chains are coloured: α1 = green, α2 = cyan, and α3 = magenta. The glycan is colored yellow. Non-carbon atoms are coloured according to the CPK convention.

Supplementary Figure 7

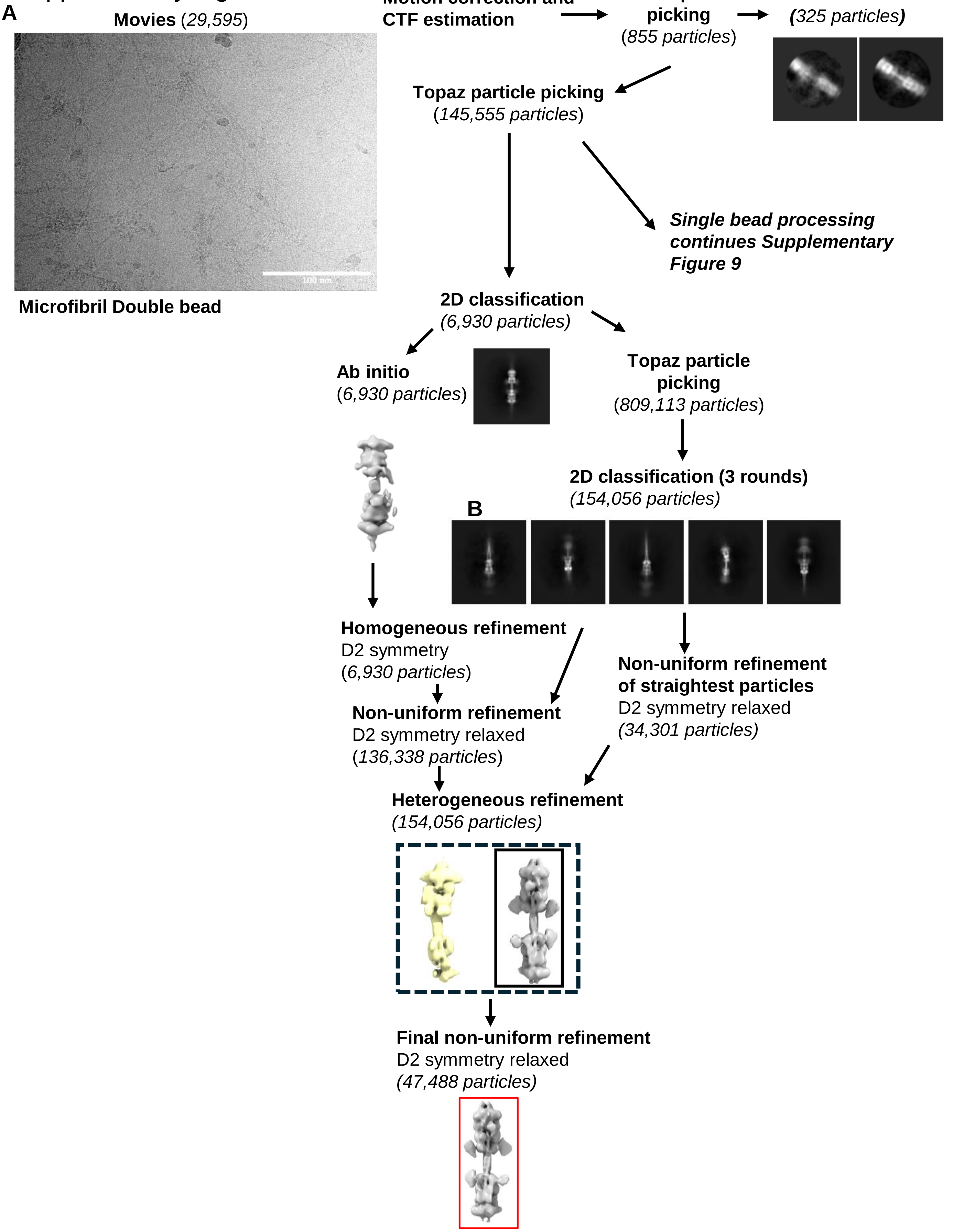


**Supplementary Figure 7: Bovine microfibril double bead single particle averaging processing workflow**

A schematic diagram of the processing of the bovine collagen VI microfibril double bead. Numbers of particles used in each step are shown. Black box highlights which 3D class was picked in heterogeneous refinement. (A) Representative cryoEM image of bovine collagen VI microfibrils. Scale bar = 100 nm. (B) Classum images of aligned collagen VI double beads. The box size is 99.5 nm x 99.5 nm. The final refinement is highlighted with a red box.

Supplementary Figure 8

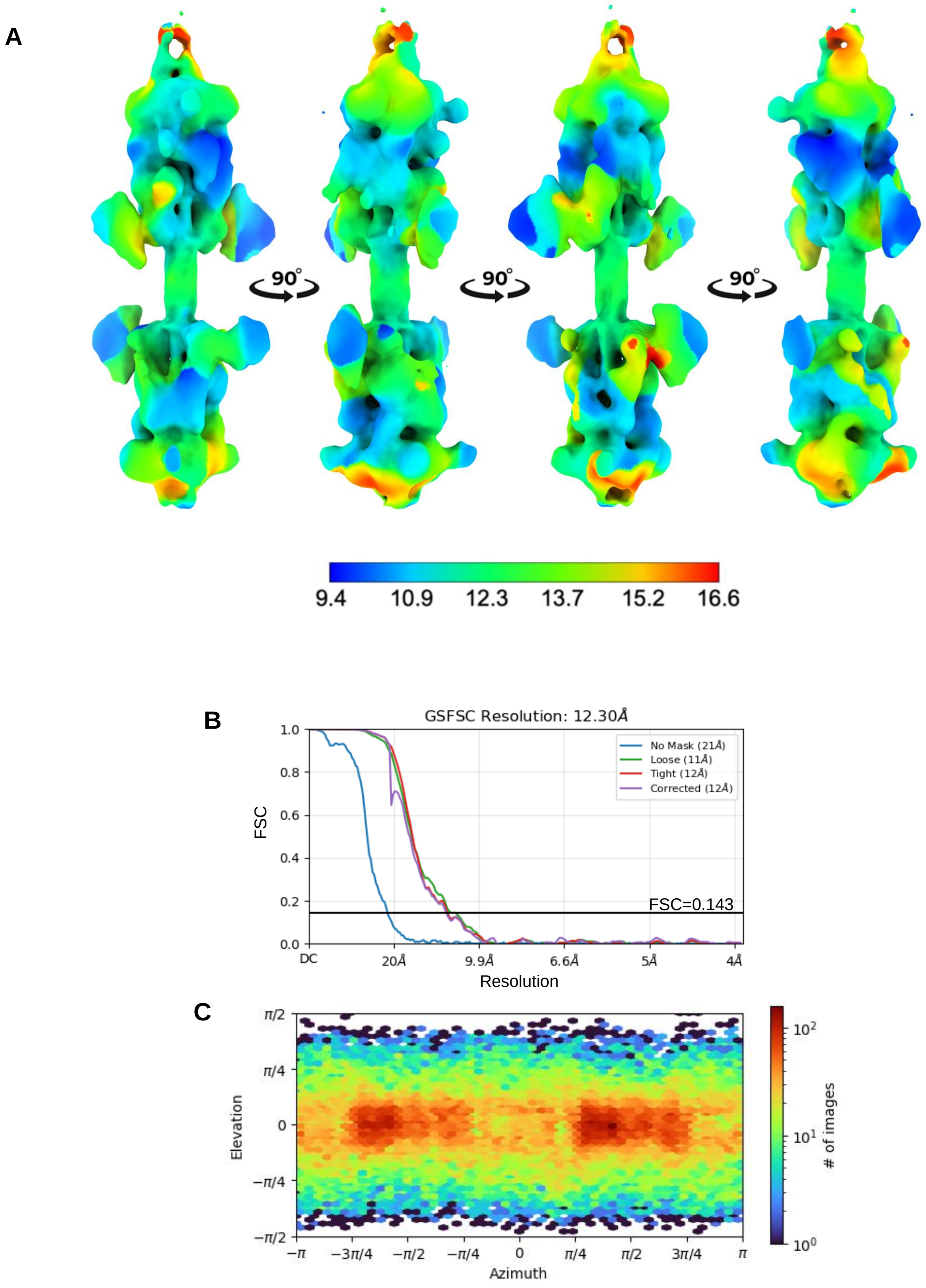


**Supplementary Figure 8: CryoEM analysis of bovine collagen VI microfibril double bead region** (A) CryoEM density map of the collagen VI double bead region coloured by local resolution (the scale is given in Angstroms). (B) A plot of the FSC between two independently refined half-maps of the collagen VI double bead. The final map was reconstructed from particles which were extracted in a 1200 x 1200 pixel box and were down-sampled to 512 x 512 pixels with a pixel size of 1.94 Å/pixel. The resolution is 12.30 Å at a cutoff of 0.143. Source data are provided as a Source Data file. (C) The viewing direction distribution plot of the aligned particles in the final dataset.

Supplementary Figure 9

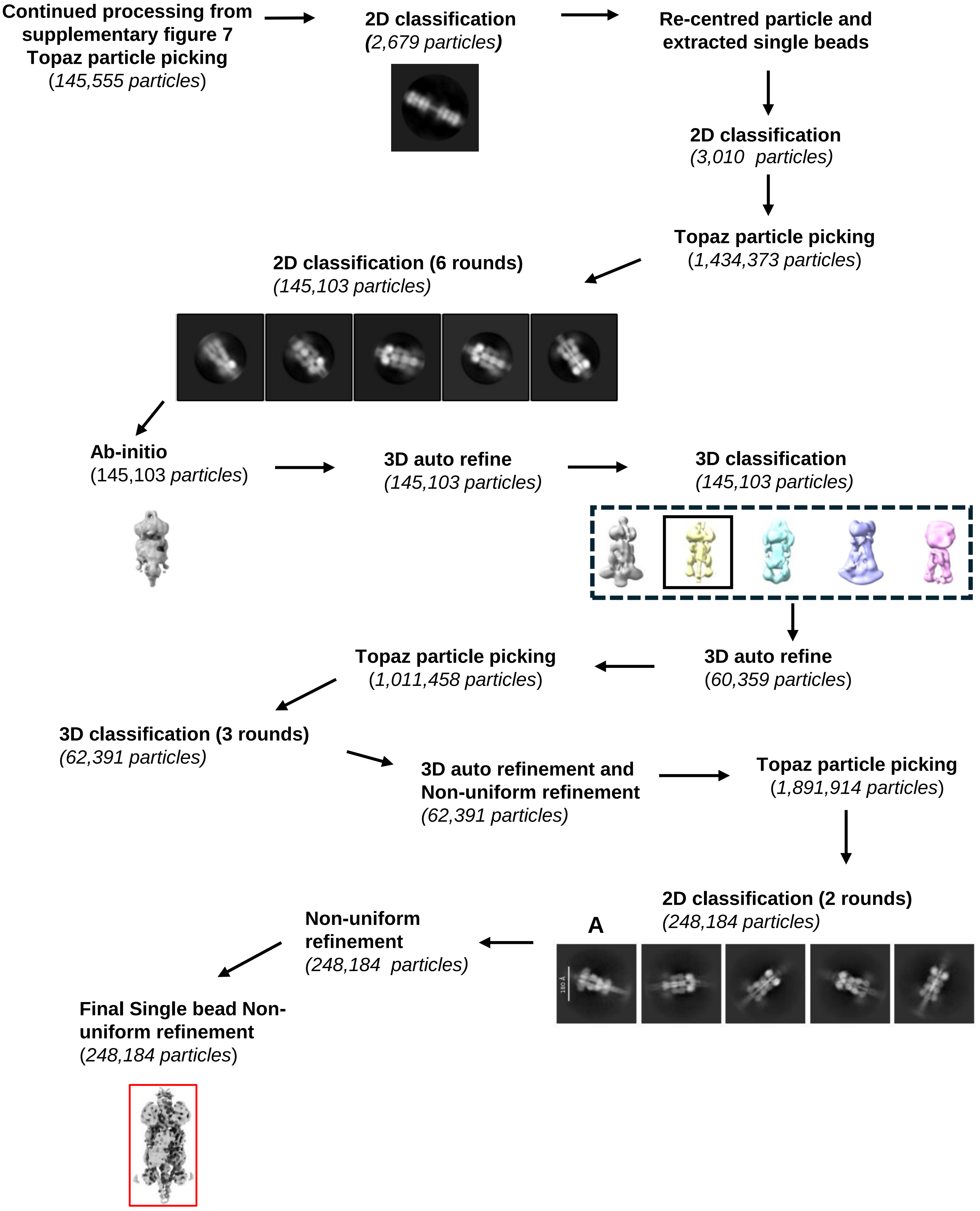


**Supplementary Figure 9: Bovine microfibril bead single particle averaging processing workflow**
A schematic diagram of the processing of the single bead of bovine collagen VI microfibrils. Numbers of particles used in each step are shown. Black box highlights which 3D class was picked in 3D classification. (A) Classum images of aligned collagen VI beads. The box size is 42.4 nm x 42.4 nm. The final refinement is highlighted with a red box. All refinements had C2 symmetry applied.

Supplementary Figure 10

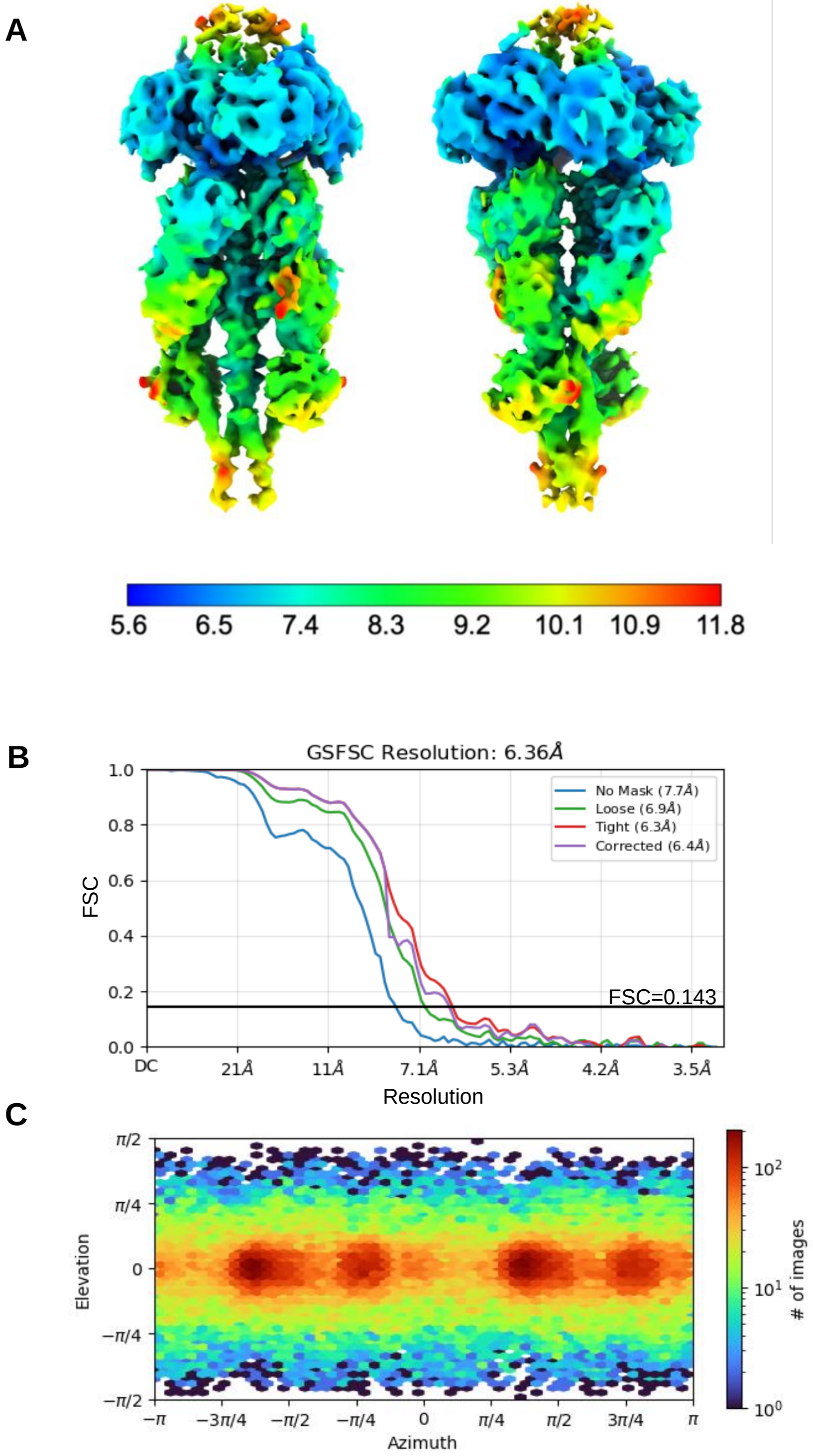


**Supplementary Figure 10: CryoEM of the single bead of bovine collagen VI microfibrils**
(A) CryoEM density map of the collagen VI bead region coloured by local resolution (the scale is given in Angstroms). (B) A plot of the FSC between two independently refined half-maps of the collagen VI bead. The final map was reconstructed from particles which were extracted using a 512 x 512 pixel box and were down-sampled to 256 x 256 pixels to give a pixel sampling of 1.65 Å/pixel. The resolution is 6.36 Å at a cutoff of 0.143. Source data are provided as a Source Data file. (C) The viewing direction distribution plot of the aligned particles in the final dataset.

Supplementary Figure 11

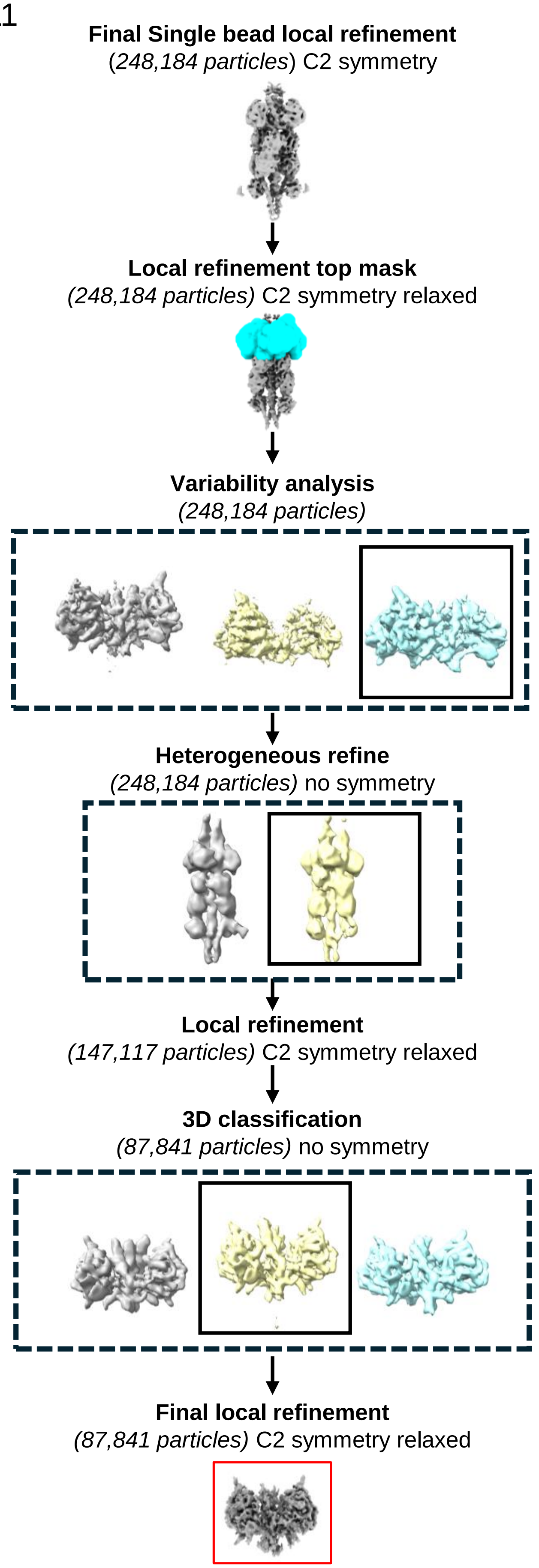


**Supplementary Figure 11: CryoEM data processing workflow for the local refinement of the top of the bovine collagen VI microfibril bead region**

A schematic diagram of the local refinement of the head part of the bovine collagen VI microfibril bead. Numbers of particles used in each step are shown. Black boxes highlight which 3D class or cluster was picked in 3D variability, heterogeneous refinement or 3D classification jobs. The final refinement is highlighted with a red box.

Supplementary Figure 12

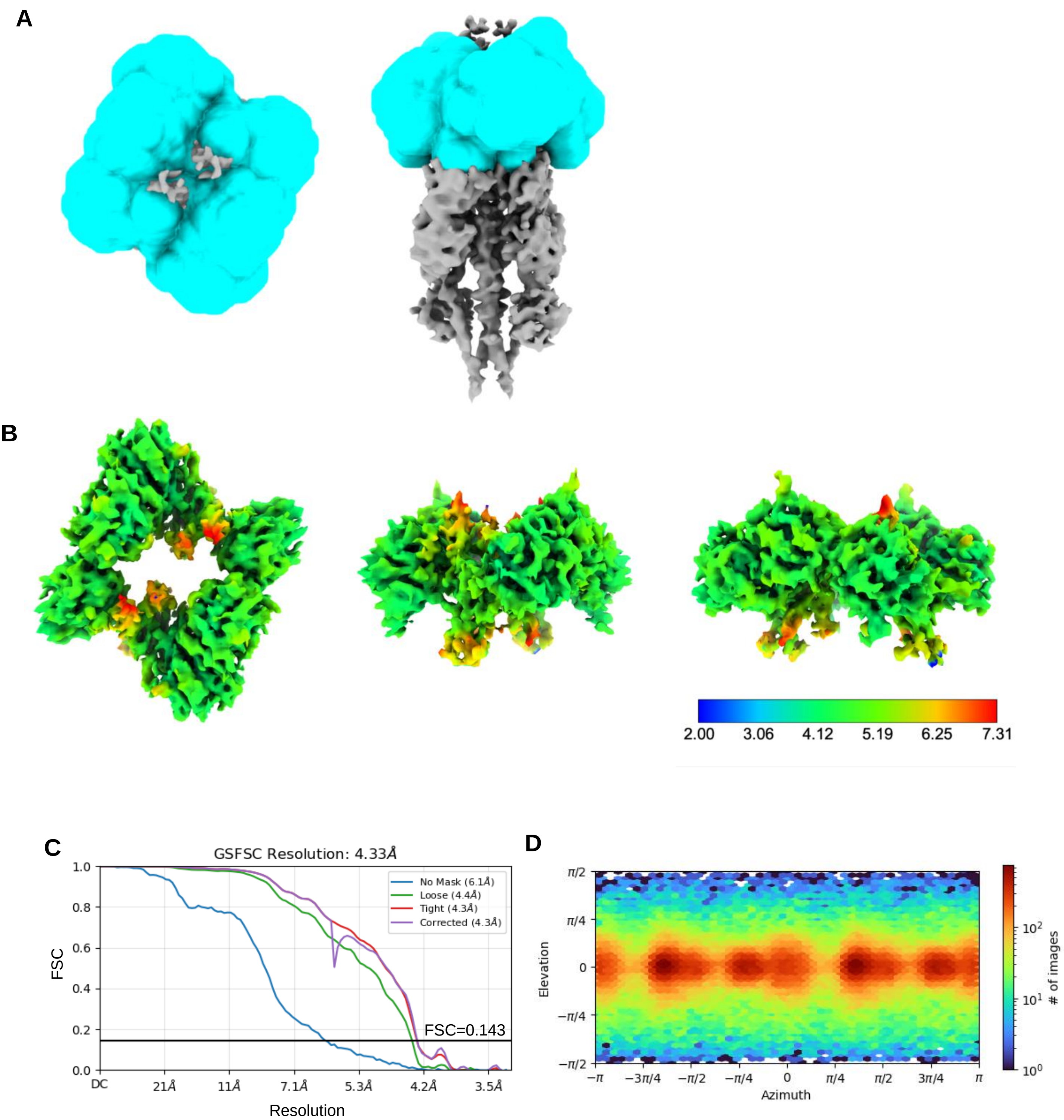


**Supplementary Figure 12: Local refinement of the top of the single bead of bovine collagen VI microfibrils**

(A) CryoEM density map of the bovine collagen VI microfibril single bead with an overlaid binary mask which was used for local refinement of the top region of the bead. (B) Electron density map of the locally refined top region of the collagen VI bead region coloured by local resolution. The scale is given in Angstroms. (C) A plot of the FSC between two independently refined half-maps of this region, extending out to the Nyquist frequency of 3.3 Å. The final map was reconstructed from particles which were extracted using a 512 x 512 pixel box and were down-sampled to 256 x 256 pixels to give a pixel sampling of 1.65 Å/pixel. The resolution is 4.33 Å at a cutoff of 0.143. Source data are provided as a Source Data file. (D) The viewing direction distribution plot of the aligned particles in the final dataset.

# Supplementary Figure 13

A

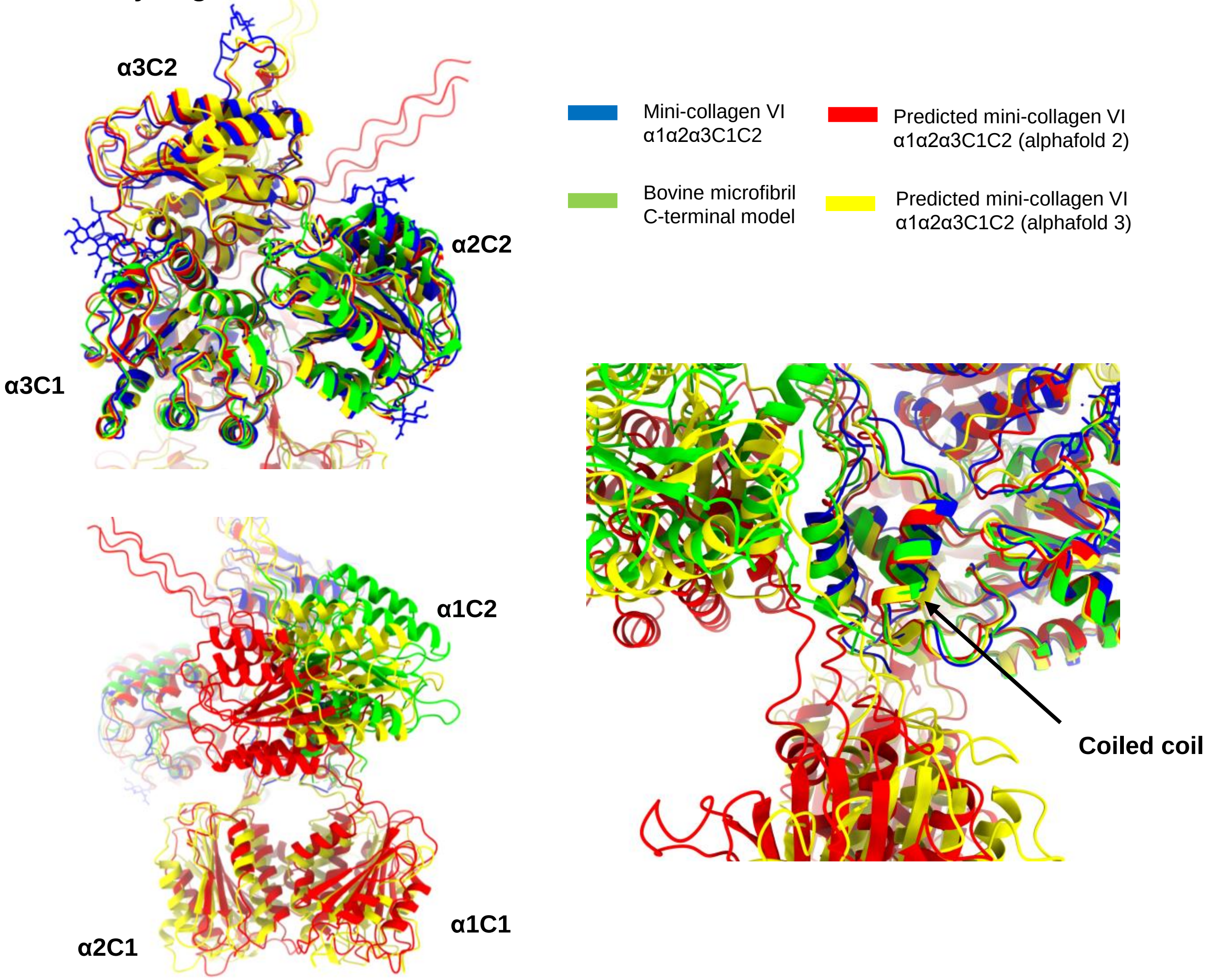


B

| | Predicted mini-collagen VI α1α2α3$^{C1C2}$ (AlphaFold 2) | Predicted mini-collagen VI α1α2α3$^{C1C2}$ (AlphaFold 3) | CryoEM of mini-collagen VI α1α2α3$^{C1C2}$ | Bovine microfibril C-terminal cryoEM model |
|---|---|---|---|---|
| Predicted mini-collagen VI α1α2α3$^{C1C2}$ (AlphaFold 2) | - | 3.288 Å (717 residues) | 1.12 Å (439 residues) | 1.587 Å (251 residues) |
| Predicted mini-collagen VI α1α2α3$^{C1C2}$ (AlphaFold 3) | 3.288 Å (717 residues) | - | 1.311 Å (666 residues) | 1.92 Å (583 residues) |
| CryoEM of mini-collagen VI α1α2α3$^{C1C2}$ | 1.12 Å (439 residues) | 1.311 Å (666 residues) | - | 1.335 Å (442 residues) |
| Bovine microfibril C-terminal cryoEM model | 1.587 Å (251 residues) | 1.92 Å (583 residues) | 1.335 Å (442 residues) | - |

**Supplementary Figure 13: Comparison of collagen VI atomic models.**
(A) Shown are overlapping cartoon representations of the heterotrimeric mini collagen VI α1α2α3$^{C1C2}$ from cryoEM data (blue), the predicted α1α2α3$^{C1C2}$ model from AlphaFold2 (red), the predicted α1α2α3$^{C1C2}$ model from AlphaFold3 (yellow) and the bovine collagen VI microfibril C-terminal cryoEM atomic model (green). AlphaFold 3 was released following model building so comparisons were made post-hoc using the model shown in Supplementary Figure 16. (B) A table of pairwise calculated RMSD values in Å for aligned atomic models. Numbers of amino acid residues used in the comparison are shown in brackets, calculated with GESAMT (Krissinel, J Mol Biochem (2012) 1:76-85).

Supplementary Figure 14

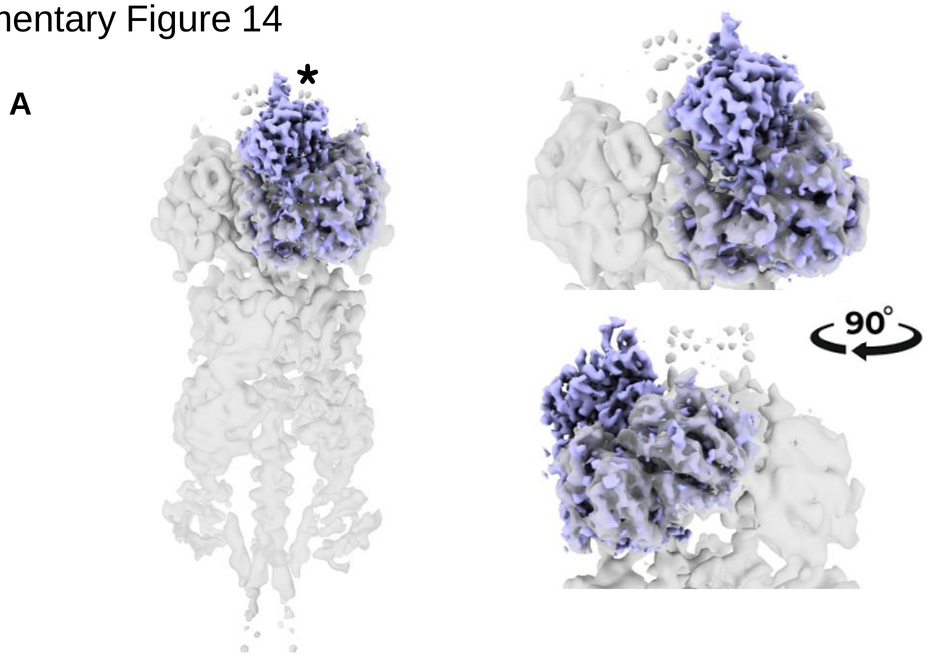


**Supplementary Figure 14: Correlation of mini-collagen VI map with microfibril map and docking C1 and N1 domains into microfibril bead.** (A) Mini-collagen VI α1α2α3$^{C1C2}$ cryoEM map shown in purple was docked into the bovine single bead map using Chimera fit in map, showing the densities compare favourably. The fit included the additional density in the mini-collagen VI α1α2α3$^{C1C2}$ map for the (α3)C2 domain (indicated by an asterisk), which is not present in the mature microfibril (processed by furin in microfibril maturation), and had a correlation = 0.6569. Both maps were Fourier filtered to 6.4 Å resolution before docking in ChimeraX was performed. All correlations were carried out at a map threshold level of 0.86, which is 7.4 standard deviations above the mean, as used for the isosurfaces shown.

Supplementary Figure 15

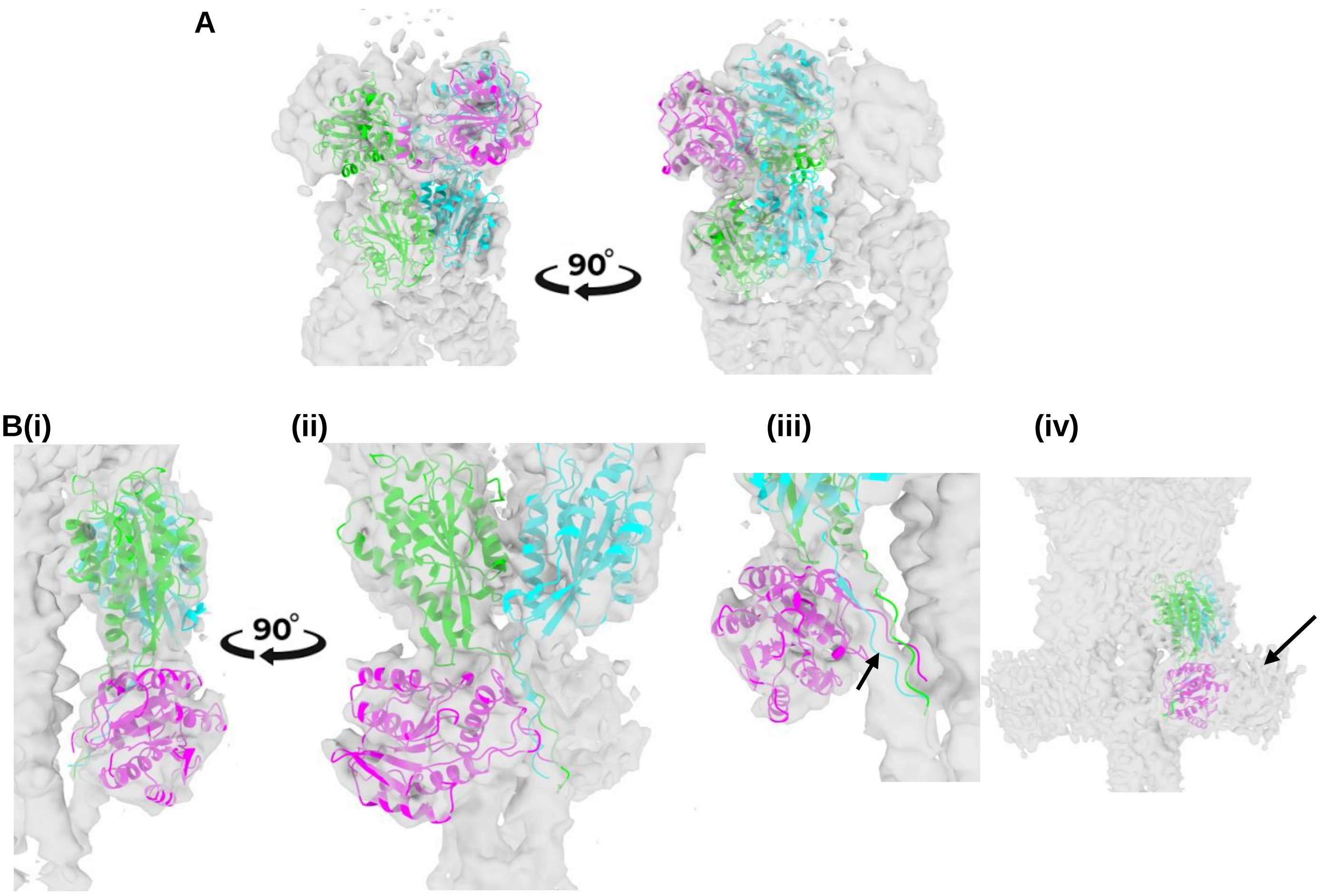


**Supplementary Figure 15: Docking C1 and N1 domains into the microfibril bead map.** (A) The AlphaFold2 $\alpha1\alpha2\alpha3^{C1C2}$ predicted model with (α)C2 domain removed, rigidly docked into the single bead using a map simulated from atoms. Correlation = 0.6273, reflecting that though the AlphaFold2 model is a good fit, the exact relative orientation of the domains does not exactly match the experimental EM map. (Bi and ii) AlphaFold3 model of the bovine $\alpha1^{N1}\alpha2^{N1}\alpha3^{N1}$ and the first 9 residues of the collagenous region rigidly docked into the single bead with a correlation = 0.8877, shown in two perpendicular views. The higher correlation indicates that the relative orientations of the N domains are accurately modelled in this case. (Biii) Highlighted by black arrow is the N-terminal end of the collagenous region. (Biv) When the map is rendered at a lower threshold value (0.4), density can be seen next to the (α3)N1 domain, highlighted by a black arrow, corresponding to the flexible α3 N2-N9 region. All correlations were carried out at a map threshold level of 0.86, which is 7.4 standard deviations above the mean, as used for the isosurfaces shown in A and B. To compare the predicted atomic models with the experimental EM map, ChimeraX was used to create a 6.4 Å density map from the atomic models (to match the map resolution), which was then docked to the EM map.

Supplementary Figure 16

A

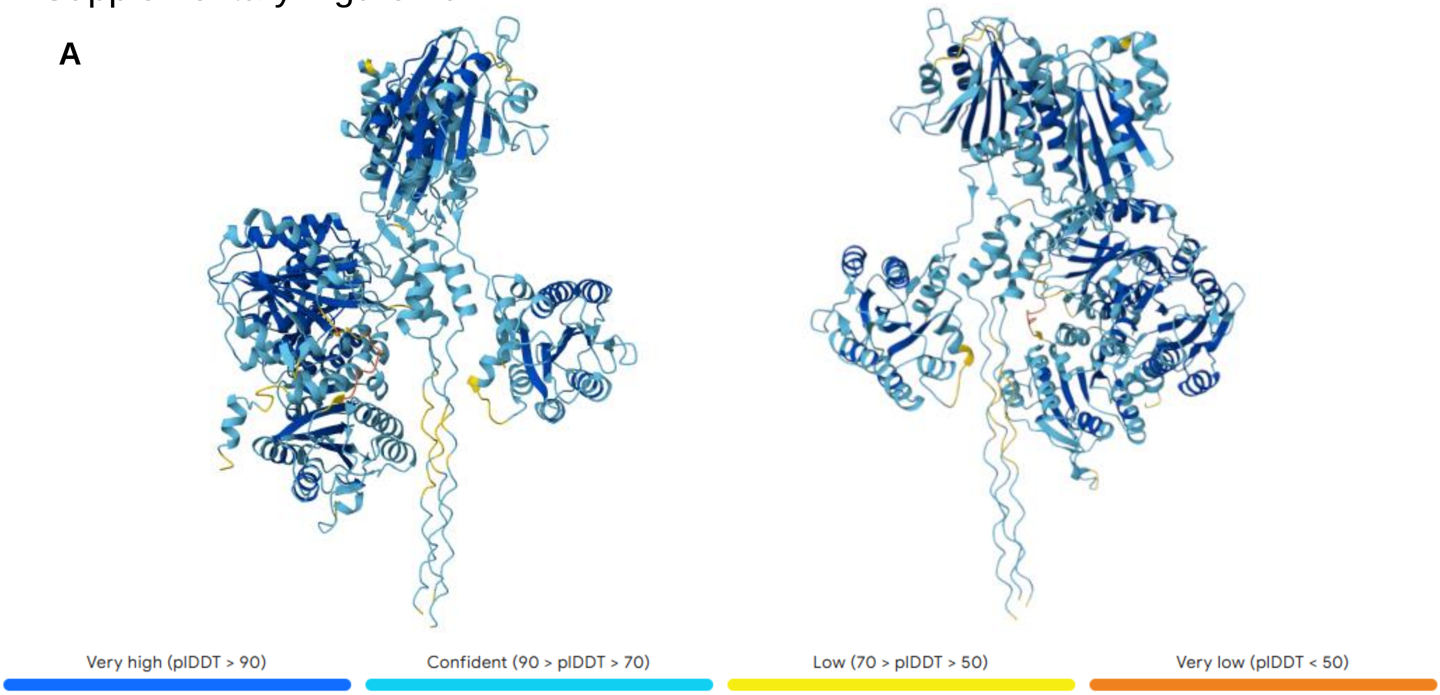


B

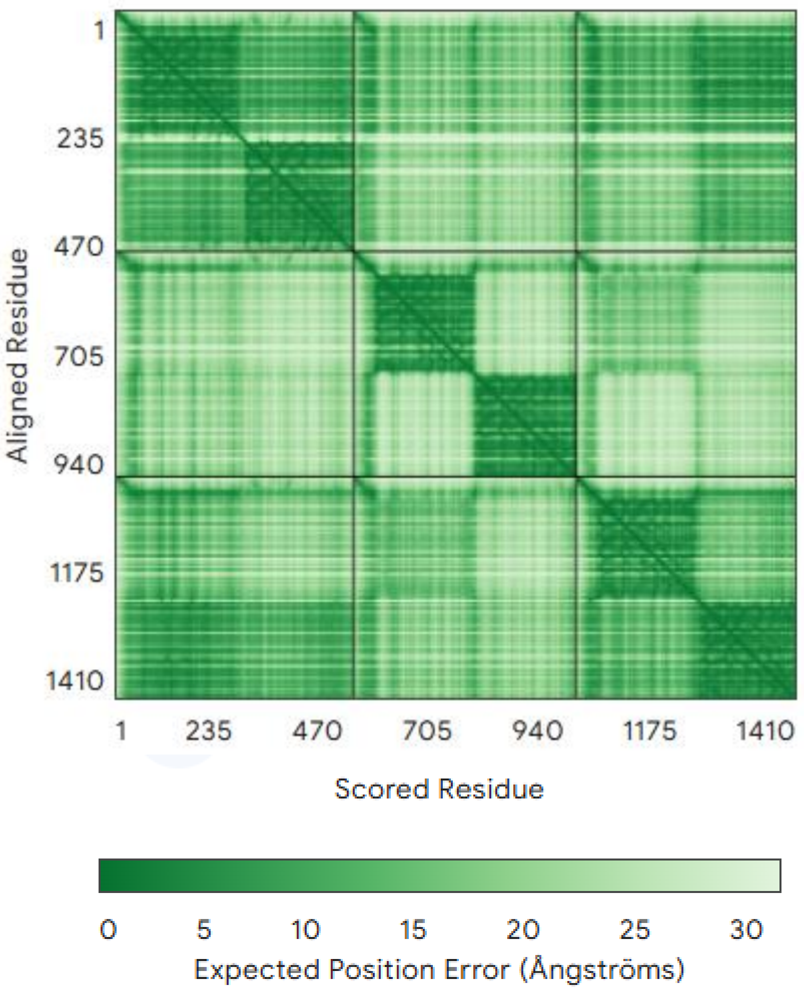


**Supplementary Figure 16: AlphaFold 3 prediction of heterotrimeric mini collagen VI α1α2α3C1C2 construct** (A) An AlphaFold 3 model of the human α1α2α3C1C2 construct shown in two orientations, with ipTM of 0.68 and pTM of 0.72. Uniprot sequence human COL6A1 P12109 residues 566-1028; human COL6A2 P12110 residues 564-1019; human COL6A3 P12111 residues 2347-2840. The model is coloured by plDDT score. (B) Predicted alignment error plot coloured by expected position error (Ängströms).

Supplementary Figure 17

A

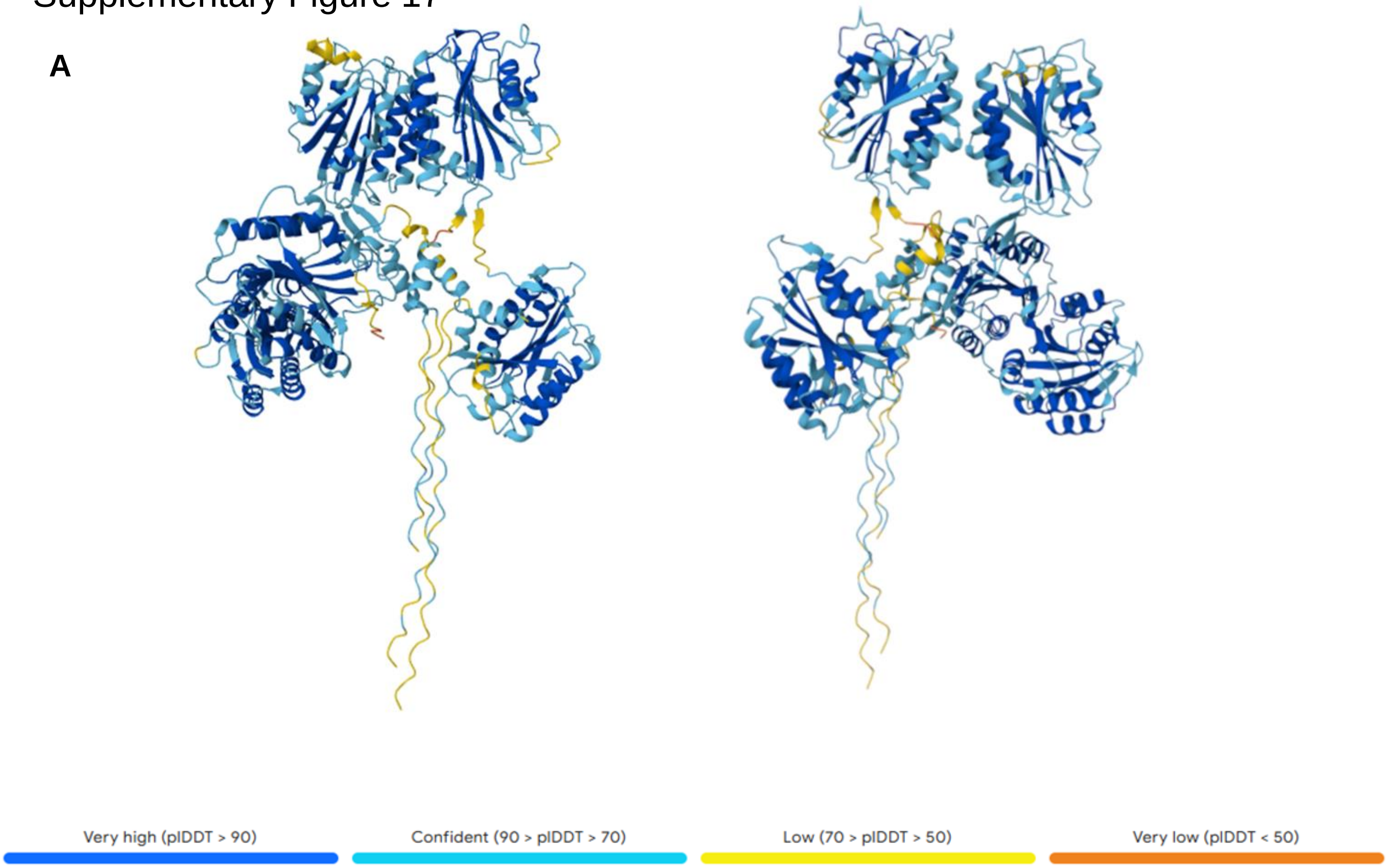


B

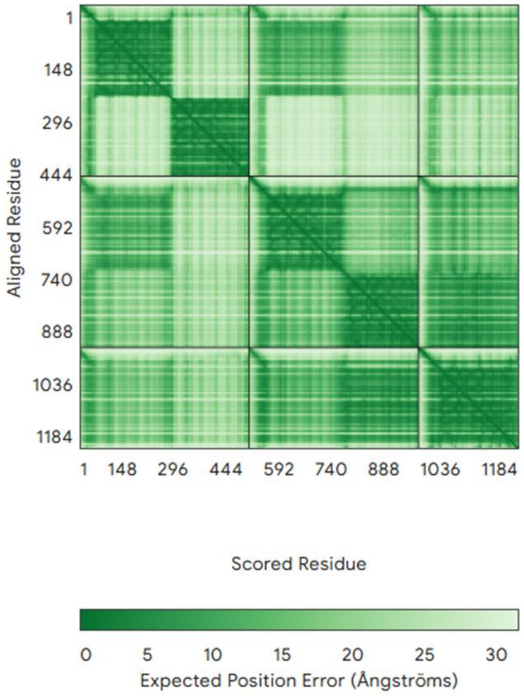


**Supplementary Figure 17: AlphaFold 3 prediction of heterotrimeric bovine C-terminal region of collagen VI** (A) An AlphaFold 3 model of the heterotrimeric bovine C-terminal region of collagen VI including the C1 and C2 domains, with ipTM of 0.60 and pTM of 0.66. Uniprot sequence bovine COL6A1 E1BI98 residues 572-1027; bovine COL6A2 A0AAA9TXG2 residues 562-1027; bovine COL6A3 A0AAA9TAB4 residues 2171-2439. The model is coloured by plDDT score. (B) Predicted alignment error plot coloured by expected position error (Ängströms).

Supplementary Figure 18

A

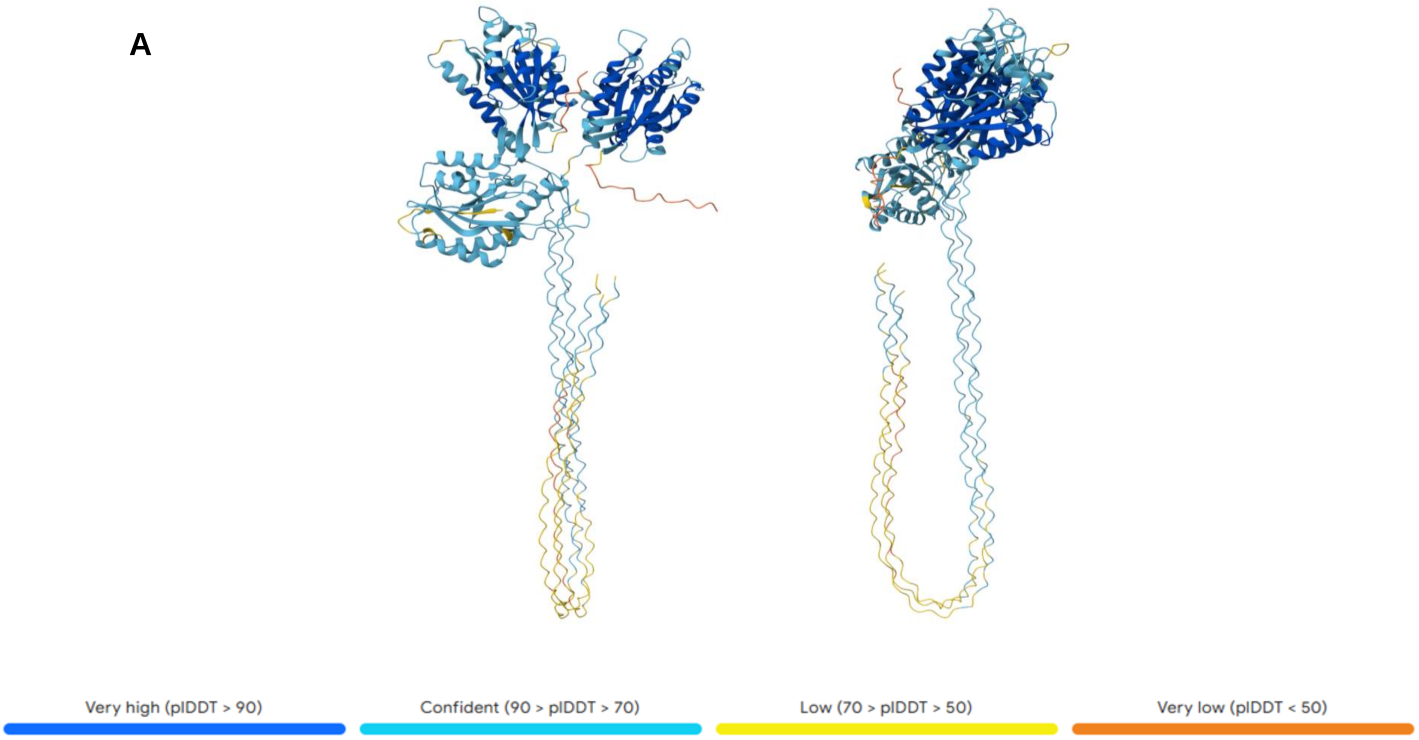


B

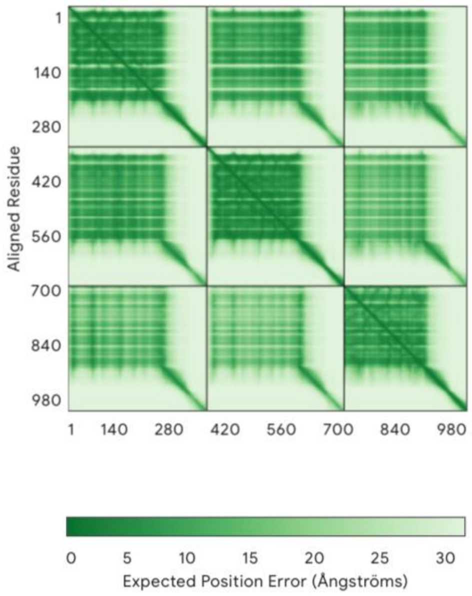


**Supplementary Figure 18: Alphafold 3 prediction of the $\alpha1^{N1}\alpha2^{N1}\alpha3^{N1}$ and 100 residues of the collagenous region of bovine collagen VI**

(A) AlphaFold 3 model of the bovine $\alpha1^{N1}\alpha2^{N1}\alpha3^{N1}$ and the first 100 residues of the collagenous region. Uniprot sequence bovine COL6A1 E1BI98 residues 21-361; bovine COL6A2 A0AAA9TXG2 residues 31-369; bovine COL6A3 A0AAA9TAB4 residues 1668-1970 with an ipTM of 0.53 and pTM of 0.58. The model is coloured by plDDT score. (B) Predicted alignment error plot coloured by expected position error (Ängströms).

Supplementary Table 1

**BioSAXS data collection of ColVIα1α2α3$^{C1C2}$**

| | ColVIα1α2α3$^{C1C2}$ |
|---|---|
| **Sample parameters** | |
| Molecular weight [kDa] | 158.9 |
| Concentration [mg/ml] | 1.0 |
| Buffer | 20 mM HEPES, 500 mM NaCl, 2mM $CaCl_2$, pH 7.4 |
| **Beamline parameters** | |
| Synchrotron | Diamond Light Source, Oxford, U.K. |
| Beamline | B21 |
| Wavelength [Å] | 0.9464 |
| Energy [keV] | 13.1 |
| Flux [photons/s] | $4 \times 10^{12}$ |
| Detector | Dectris EigerX 4M |
| Detector distance [m] | 3.722 |
| Q range [$Å^{-1}$] | 0.0045 – 0.34 |
| Exposure temperature [$^{o}$C] | 15 |
| Exposure time [s] | 20 × 1 |
| Collection mode | Batch mode |
| **Data processing** | |
| Data reduction | DAWN |
| Data analysis | ScÅtter IV |
| $I(0)$ [$cm^{-1}$] | 0.172 |
| $R_g$ [Å] | 47.7 |
| $D_{max}$ [Å] | 145 |
| Porod Exponent $P_E$ | 3.879 |
| **FoXS analysis** | |
| PDB | AlphaFold |
| $\chi^2$ | 1.76 |

Supplementary Table 2

**Supplementary Table 2: Cryo-EM data collection, refinement and validation statistics**

| | α1α2α3[C1C2] heterotrimer (EMD-51567) (PDB 9GTU) | C-terminal bead region (EMD-51984) (PDB 9HAN) | Single bead (EMD-52366) | Double bead (EMD-52362) |
|---|---|---|---|---|
| **Data collection and processing** | | | | |
| Magnification | 130,000 | 105,000 | 105,000 | 105,000 |
| Voltage (kV) | 300 | 300 | 300 | 300 |
| Electron exposure (e–/Å$^2$) | 28.11 | 50.98 | 50.98 | 50.98 |
| Defocus range (μm) | -2 - -0.75 | -2 - -0.75 | -2 - -0.75 | -2 - -0.75 |
| Pixel size (Å) | 0.651 | 0.829 | 0.829 | 0.829 |
| Symmetry imposed | C1 | C1 | C2 | C1 |
| Initial particle images (no.) | 10665534 | 1011458 | 1434373 | 809113 |
| Final particle images (no.) | 246984 | 87841 | 248184 | 47488 |
| Map resolution (Å) FSC threshold | 3.14 (0.143) | 4.33 (0.143) | 6.36 (0.143) | 12.3 (0.143) |
| Map resolution range (Å) | 2.916 - 8.416 | 3.669 - 8.776 | 5.611 - 25.267 | 9.407 - 18.551 |
| **Refinement** | | | | |
| Initial model used (PDB code) | - | - | | |
| Model resolution (Å) FSC threshold | 3.4 (0.0/0.143/0.5) | 4.4 (0.0/0.143/0.5) | | |
| Model resolution range (Å) | 3.0/3.1/3.4 | 4.1/4.2/4.4 | | |
| Map sharpening *B* factor (Å$^2$) | 86.5 | 175.1 | | |
| Model composition | | | | |
| Non-hydrogen atoms | 5613 | 11143 | | |
| Protein residues | 706 | 1414 | | |
| Ligands | 8 | 17 | | |
| *B* factors (Å$^2$) | | | | |
| Protein | 52.67 | 92.17 | | |
| Ligand | 97.00 | 102.2 | | |
| R.m.s. deviations | | | | |
| Bond lengths (Å) | 0.003 | 0.003 | | |
| Bond angles (°) | 0.543 | 0.574 | | |
| Validation | | | | |
| MolProbity score | 2.05 | 2.24 | | |
| Clashscore | 10 | 16.1 | | |
| Poor rotamers (%) | 0.66 | 0.08 | | |
| Ramachandran plot | | | | |
| Favored (%) | 91.4 | 90.67 | | |
| Allowed (%) | 8.45 | 9.33 | | |
| Disallowed (%) | 0.14 | 0 | | |

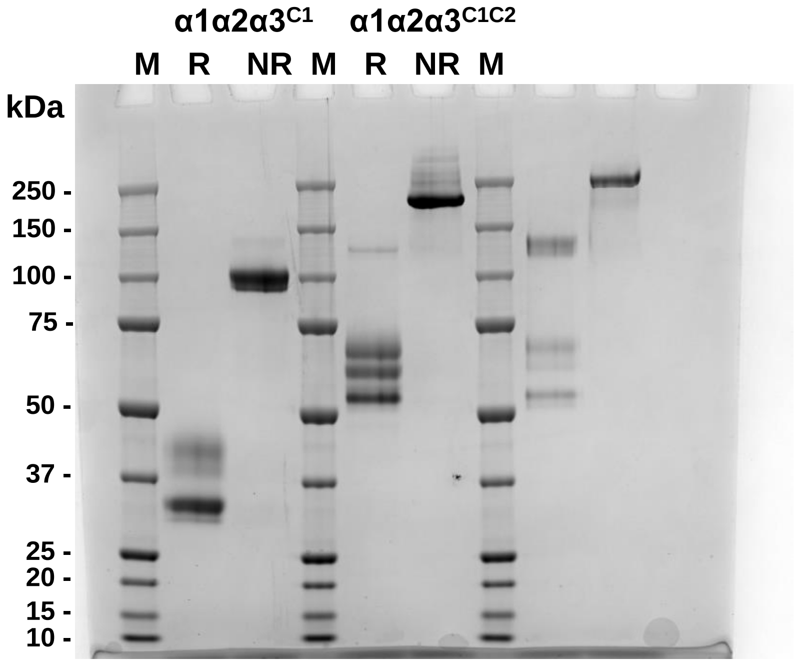


Uncropped SDS-PAGE gel used in main manuscript figures 2b and 5b showing the α1α2α3$^{C1C2}$ and α1α2α3$^{C1}$, respectively. The unlabelled lanes on the right hand side of the gel relate to a sample that was not described in the manuscript.

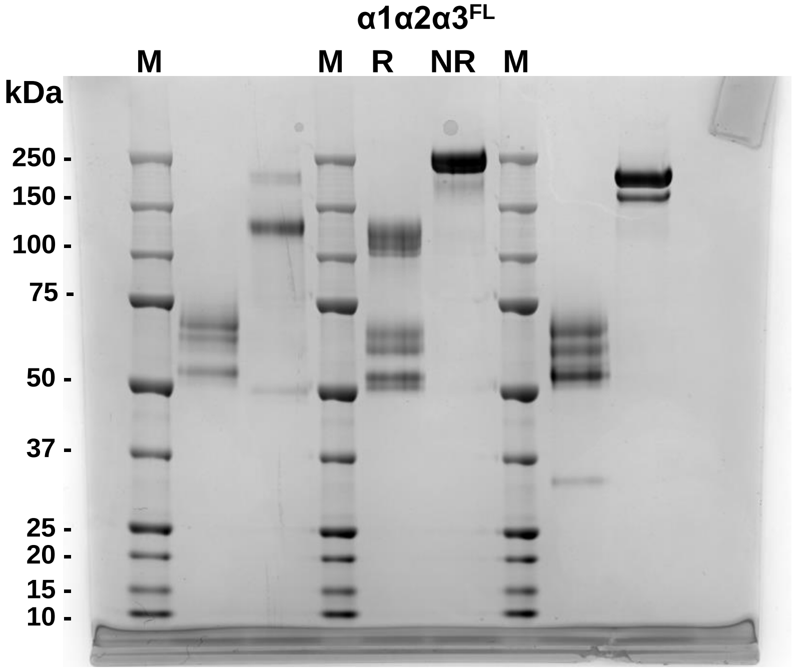


Uncropped SDS-PAGE gel from supplementary figure 1B showing the α1α2α3$^{FL}$ The unlabelled lanes on the right and left hand sides of the gel relate to samples that were not described in the manuscript.

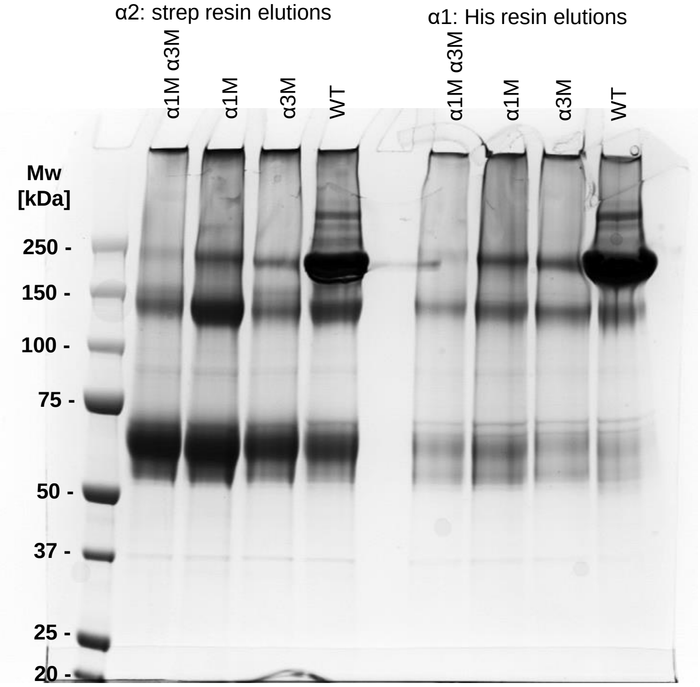


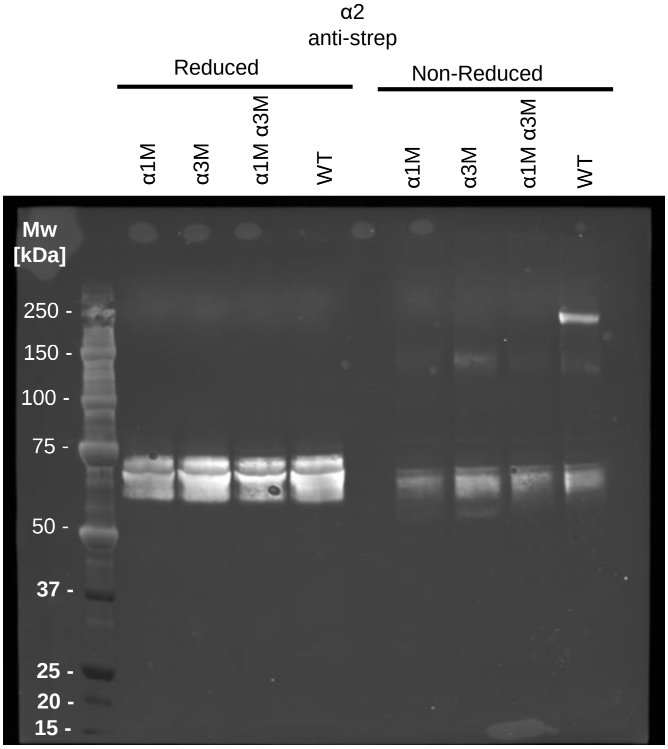


**Uncropped SDS-PAGE gel and western blot show in main manuscript Figure 5f(i) and (ii), respectively.**
