# Supplementary material for: Collagen VI microfibril structure reveals mechanism for molecular assembly and clustering of inherited pathogenic mutations
Source: Nat Commun. 2025 Aug 14;16:7549. doi: 10.1038/s41467-025-62923-3 (PMC12354898; doi:10.1038/s41467-025-62923-3)
Supplement: Supplementary file 2 — Reporting Summary [file 41467_2025_62923_MOESM2_ESM.pdf]

nature portfolio

Corresponding author(s): Clair Baldock

Last updated by author(s): Jun 19, 2025

Reporting Summary

Nature Portfolio wishes to improve the reproducibility of the work that we publish. This form provides structure for consistency and transparency in reporting. For further information on Nature Portfolio policies, see our Editorial Policies and the Editorial Policy Checklist.

Statistics

For all statistical analyses, confirm that the following items are present in the figure legend, table legend, main text, or Methods section.

n/a	Confirmed	
☐	☒	The exact sample size (*n*) for each experimental group/condition, given as a discrete number and unit of measurement
☐	☒	A statement on whether measurements were taken from distinct samples or whether the same sample was measured repeatedly
☒	☐	The statistical test(s) used AND whether they are one- or two-sided<br>*Only common tests should be described solely by name; describe more complex techniques in the Methods section.*
☒	☐	A description of all covariates tested
☒	☐	A description of any assumptions or corrections, such as tests of normality and adjustment for multiple comparisons
☒	☐	A full description of the statistical parameters including central tendency (e.g. means) or other basic estimates (e.g. regression coefficient) AND variation (e.g. standard deviation) or associated estimates of uncertainty (e.g. confidence intervals)
☒	☐	For null hypothesis testing, the test statistic (e.g. *F*, *t*, *r*) with confidence intervals, effect sizes, degrees of freedom and *P* value noted<br>*Give P values as exact values whenever suitable.*
☒	☐	For Bayesian analysis, information on the choice of priors and Markov chain Monte Carlo settings
☒	☐	For hierarchical and complex designs, identification of the appropriate level for tests and full reporting of outcomes
☒	☐	Estimates of effect sizes (e.g. Cohen's *d*, Pearson's *r*), indicating how they were calculated

Our web collection on statistics for biologists contains articles on many of the points above.

Software and code

Policy information about availability of computer code

Data collection	CryoEM data - EPU (Thermofisher)<br>SEC-MALS - Astra 6.1 (Wyatt Technologies)<br>Mass photometry - DiscoverMP 2.3 (Refeyn).
Data analysis	CryoEM data analysis - Relion4.0, MotionCor2 v1.2.0, Topaz, cryoSPARC v2.<br>Structure refinement - COOT and Phenix real-space-refine.<br>Structure visualisation - UCSF ChimeraX 1.7, Pymol.<br>Structure validation - Molprobity 4.5.2.<br>Structure prediction - AlphaFold version 2.1.1. AlphaFold server (AlphaFold3)<br>SAXS analysis - ScAtter version 4; ATSAS online, FoxS webserver.<br>Visualisation of graphs - Prism version 10.3.0 (Graphpad)<br>A custom python script for visualisation of PAE plots generated by AlphaFold2 is freely available at https://doi.org/10.5281/zenodo.15880546 as indicated in the code availability statement.

For manuscripts utilizing custom algorithms or software that are central to the research but not yet described in published literature, software must be made available to editors and reviewers. We strongly encourage code deposition in a community repository (e.g. GitHub). See the Nature Portfolio guidelines for submitting code & software for further information.

Data

Policy information about availability of data

All manuscripts must include a data availability statement. This statement should provide the following information, where applicable:

- Accession codes, unique identifiers, or web links for publicly available datasets
- A description of any restrictions on data availability
- For clinical datasets or third party data, please ensure that the statement adheres to our policy

The structures and cryoEM data have been deposited to EMDB and PDB databanks for ColVIα1α2α3C1C2 and the collagen VI microfibril with accession codes PDB ID 9GTU; EMD-51567 and PDB ID 9HAN; EMD-51984, respectively. The cryoEM maps for the microfibril double and single beads have been deposited to EMDB with accession codes EMD-52362 and EMD-52366, respectively. SAXS data for ColVIα1α2α3C1C2 has been deposited in SASDBD with accession code SASDWL2.

Research involving human participants, their data, or biological material

Policy information about studies with human participants or human data. See also policy information about sex, gender (identity/presentation), and sexual orientation and race, ethnicity and racism.

Reporting on sex and gender	N/A
Reporting on race, ethnicity, or other socially relevant groupings	N/A
Population characteristics	N/A
Recruitment	N/A
Ethics oversight	N/A

Note that full information on the approval of the study protocol must also be provided in the manuscript.

Field-specific reporting

Please select the one below that is the best fit for your research. If you are not sure, read the appropriate sections before making your selection.

☒ Life sciences ☐ Behavioural & social sciences ☐ Ecological, evolutionary & environmental sciences

For a reference copy of the document with all sections, see nature.com/documents/nr-reporting-summary-flat.pdf

Life sciences study design

All studies must disclose on these points even when the disclosure is negative.

Sample size	No sample size calculations were performed. For cryoEM imaging of bovine microfibrils 29,595 movies were collected and for the mini-collagen VI construct 35,706 movies were collected.
Data exclusions	The cryoEM pipeline excludes "bad" particles in an automated software pipeline that is widely utilised in the field and is well-described. For all other data types, no data were excluded from analysis.
Replication	For cryoEM, more than six microfibril purifications were performed on six different cornea samples (biological replicates) for optimisation of grid preparation and data collection strategy.
Randomization	Randomization is not relevant for this study, as there were no groups allocated in any of the experiments.
Blinding	Blinding was not relevant for the cryoEM data collection as data was collected on only one sample type in each data collection.

Reporting for specific materials, systems and methods

We require information from authors about some types of materials, experimental systems and methods used in many studies. Here, indicate whether each material, system or method listed is relevant to your study. If you are not sure if a list item applies to your research, read the appropriate section before selecting a response.

Materials & experimental systems

n/a	Involved in the study
☐	☒ Antibodies
☐	☒ Eukaryotic cell lines
☒	☐ Palaeontology and archaeology
☐	☒ Animals and other organisms
☒	☐ Clinical data
☒	☐ Dual use research of concern
☒	☐ Plants

Methods

n/a	Involved in the study
☒	☐ ChIP-seq
☒	☐ Flow cytometry
☒	☐ MRI-based neuroimaging

Antibodies

Antibodies used	Monoclonal antibody to StrepII tag.<br>IBA Life Sciences, Code : 2-1507-001<br>Dilution 1:1000
Validation	https://www.iba-lifesciences.com/strepmab-classic/2-1507-001

Eukaryotic cell lines

Policy information about cell lines and Sex and Gender in Research

Cell line source(s)	ThermoFisher scientific Expi293F human cells
Authentication	Commercial cell line, not authenticated
Mycoplasma contamination	Cells are mycoplasma free and routinely tested for mycoplasma contamination.
Commonly misidentified lines<br>(See ICLAC register)	N/A

Animals and other research organisms

Policy information about studies involving animals; ARRIVE guidelines recommended for reporting animal research, and Sex and Gender in Research

Laboratory animals	The study did not involve lab animals but bovine corneas were collected from a local abattoir.
Wild animals	The study did not involve the observation or use of wild animals.
Reporting on sex	Data on sex was not captured.
Field-collected samples	Bovine corneas were collected from a local abattoir as a waste product of the food chain.
Ethics oversight	Ethical approval was not required as animals were not bred, maintained nor sacrificed for research purposes.

Note that full information on the approval of the study protocol must also be provided in the manuscript.

Plants

Seed stocks	*Report on the source of all seed stocks or other plant material used. If applicable, state the seed stock centre and catalogue number. If plant specimens were collected from the field, describe the collection location, date and sampling procedures.*
Novel plant genotypes	*Describe the methods by which all novel plant genotypes were produced. This includes those generated by transgenic approaches, gene editing, chemical/radiation-based mutagenesis and hybridization. For transgenic lines, describe the transformation method, the number of independent lines analyzed and the generation upon which experiments were performed. For gene-edited lines, describe the editor used, the endogenous sequence targeted for editing, the targeting guide RNA sequence (if applicable) and how the editor was applied.*
Authentication	*Describe any authentication procedures for each seed stock used or novel genotype generated. Describe any experiments used to assess the effect of a mutation and, where applicable, how potential secondary effects (e.g. second site T-DNA insertions, mosiacism, off-target gene editing) were examined.*
